# A Lecture on Lateral Curvature of the Spine

**Published:** 1910-05-21

**Authors:** Alfred H. Tubby

**Affiliations:** Surgeon, Westminster Hospital, and to the Royal National Orthopædic Hospital.


### Hospital Clinics.

### A LECTURE ON LATERAL CURVATURE OF THE SPINE.

By ALFRED H. TUBBY, M.S.; Surgeon, Westminster Hospital, and to the Royal National Orthopædic Hospital.

(Delivered at the Polyclinic, Chenies Street, W.C., on Tuesday, February 15, 1910.)

Gentlemen,—On the last occasion I lectured here I discussed with you, up to a certain point, the subject of scoliosis in childhood. You will remember we went over the causation, the morbid changes, and the structural types. To-day I propose to deal with the methods of examination of the back, with the symptoms, and, as far as possible in the time allowed, with the treatment. When a child is brought to you with scoliosis it is essential that you should have a careful method of making an examination. And for this purpose the back should be bared completely. In the case of young children it is often advisable to have all the clothes removed. With older children a skirt is used, which is secured around the hips with an elastic band. For young adults we use, in addition, a short jacket, with the opening and the buttons behind. In any event the whole back should be bared from the neck to the gluteal cleft, and the skirt should not be higher than the tops of the great trochanters. The patient is then placed in a good light, so that you can see the back clearly. Then wait a few minutes. Do not touch the back at once, otherwise the patient does not assume the posture which is usual to her: the shoulders are braced up, and you thus get a false idea of the position of the back; and you take cognisance of the following points:—

### Some Signs on Inspection.

The first thing which strikes your eye is the outline made by the spinous processes, but, as I warned you last time, the outline of the spinous processes, or the deviation of that outline, is no measure of the amount of rotation; you estimate that by the prominence or depression of the ribs in the various regions. Then notice the position of the angles of the scapulæ. It is found that, as a rule, if the curve be right-sided above and to the left side below, the angle of the scapula on the convex side of the curve is elevated. You always speak of the convex side of the curve being the side to which the convexity points. Next observe the angles which the sides of the trunk make with the upper arm, which may be called the axillary angle. Then notice the condition of the flanks. As a rule, on the concave side of the curve the flank is sunken, and the crest of the ilium appears to be more prominent, or, as the mother often says, the child's hip is growing out: and on the convex side of the curve the flank is much flattened and fuller. Certain other subsidiary points should be noted, and one is the position in which the shoulders are carried: whether one shoulder is elevated or the other shoulder depressed. Finally, make the patient cross the arms on the chest and bend down. It has the effect of baring the posterior aspect of the ribs by drawing the scapulæ forward; and you will at once see whether there is any distortion of the posterior part of the ribs, and, therefore, whether there are evidences of rotation; because the effects of rotation are seen mostly in the posterior part of the ribs themselves. Next proceed to ascertain whether the spine is flexible or not, because upon the degree of flexibility will depend a good deal of the success of treatment, and how far you can rectify the curvature. In order to test this, make the patient bend forward and backward, as I show you, and then bend first to one side and then to the other. Then suspend the patient and see how far the curves come out. And, other things being equal, *i.e.* if the flexibility is not excessive, you may take it that the more flexible the spine is, the more it is amenable to treatment, and the more the curves will come out. If the spine is mobile, there is no osseous fixation of any part, and we can often reduce the curves when they are not due to osseous fixation.

### Pain in Lateral Curvature.

A symptom of considerable interest in these cases is pain. Scoliosis is not a disease, but a deformity. Still, in a number of patients there is a complaint of pain; which, in most instances, is due to excessive tenderness of muscles and ligaments. You will understand that the muscles and ligaments are stretched on the convex side, and on the concave side they are squeezed up. Clinically, you will find that in the commonest form of curve, the right dorsal and left lumbar, the pain is limited to a particular region, namely, just below the angle of the scapula, on the convex side of the dorsal curve. We constantly find that pain is complained of there, and I believe it is due to the stretching of the muscles and ligaments in that area. Many patients complain of abdominal pains, and they are due to alterations in the positions of the viscera, and to the dragging on their attachments. If, for instance, there is a right convex dorsal curve of any great extent, the liver is displaced, and these patients frequently complain of pain in the abdomen, owing to stretching and tension of the ligaments which support the liver. If there is a sharp bend at the dorso-lumbar junction the pain runs along the course of the last dorsal nerve, especially where the lateral branches come off and pass over the crest of the ilium. I believe the pain to be due to compression of the last dorsal nerve between the twelfth rib and the crest of the ilium. Those who see many cases of scoliosis know that in some instances the last rib and the crest of the ileum are in contact, and, as it were, grind together with every movement of the patient. So in those cases the last dorsal nerve becomes severely compressed. One instance I call to mind, and that is the case of a young girl who had severe right scoliosis and constant pain over the distribution of the last dorsal nerve in front, and, therefore, over what is known as the appendix region. And so persistent was it that a surgeon was called in and decided the removal of the appendix. But before doing so the young woman was brought to us, and I found, on suspending her, that the pain entirely disappeared. So we put her into a support in the suspended position, and she had no further trouble; and I have every reason to believe she is still in possession of her appendix.

Finally, a certain number of cases of scoliosis occur in neuralgic, hysterical women, and they complain of vague and flying pains. The severity of the pain increases with the gravity of the deformity, and many people's lives are rendered miserable by this very severe condition, so that we often have to treat them definitely for this pain.

### Brief Notes on Some Forms of Treatment.

Now we come to the question of *treatment*, which, of course, is highly important. And we may divide it into preventive and curative. Preventive treatment consists, as far as we are concerned, in carefully reviewing all the factors which we find in a case of scoliosis. Let us take them in order, beginning with small children. Firstly, rickets and rickety children should have their backs carefully examined, and if there is any evidence of excessive kyphosis, which, as I explained to you last time, is the antecedent condition in almost all these cases of lateral curvature, you must proceed to treat the kyphosis. You may do so by keeping the child recumbent: and if it is to be carried about, a simple wicker tray, shaped very much like the tray used by butchers, and well padded, is the best thing to carry it about in. Also note the position in which the nurse carries the child. Often you will see that she does so on the arm with the hand slightly raised above the level of the elbow; and the result is that the child's buttocks are on an inclined plane, which determines the direction in which the scoliosis will take place. All vicious and bad positions in schools should be corrected. I made some remarks last time on the conditions which are prone to produce bad postures. It is, above all, important that you should make it clear that a fatigued child is a child who naturally lapses into bad postures, and so every effort should be made to prevent undue fatigue, particularly by the use of suitable school desks. The old fashion was to seat children on a form without a back, and let the children loll about as they wished. But now that is altered, and suitable desks are supplied. The best *résumé* on the subject of school desks is to be found in the report of Dr. Lovett, of Boston, U.S.A., to the School Commission, and there you will find details as to the exact height of the desk and the position in which the child should sit. But there are adequate desks supplied in England, and I think the best of all are those made by the North of England School Furnishing Company; they supply desks which allow the child to sit comfortably without pain or fatigue.

### Clothing.

An important point is that concerning the clothing of children. At one time most children had their lower garments suspended around the waist. For various reasons it was thought this was harmful, and so the plan was introduced of suspending them by braces across the shoulders. But it was found by experience that the weight of heavy garments so suspended caused the shoulders to droop forward, and when the child became weary, it would have that stoop. You know that a heavy overcoat made by one tailor may not appear nearly so heavy as one of the same weight made by another. The difference depends in the one case on the weight falling near the mid line, around the neck, whereas in the other case the weight is more upon the outer part of the shoulders. The best way is to have the usual braces made but not crossed, and they are connected by a piece of webbing or cloth, just below the nape of the neck, so that the weight is borne at that spot and not upon the shoulders.

This is the secret of suspending children's garments comfortably.

### Some of the Correction Methods.

Now a few words as to the corrective treatment of spinal deformity. In the production of scoliosis, at least four factors have to be considered. The first is the assumption of abnormal postures from various causes. The second is fixation in these vicious positions, and the constant influence of such causes. The third is the continuance of such bad positions owing to the weight of the parts which have to be supported, namely, the weight of the shoulders, head and neck. Lastly, the deformity depends on the deficient tension of the longitudinal spinal muscles. We may consider what is the force which keeps the spines themselves in the upright position. The vertebral column is a mass of discs piled one above another, and they are kept in the erect position by muscular supports or stays, like those of the masts of a ship. If the tension is equal on both sides and is of the proper amount, the pile of discs is held vertically. If the tension is deficient on one side the pile of discs will tend to drop away towards the weak side. If the tension is deficient on the whole, the piled-up discs will move in certain directions, which will depend on gravity, and on the position of the base on which they rest.

### Rest.

A great part, therefore, of the treatment of scoliosis is directed towards improving the condition of the muscles. The means we have to consider are, first, those of a general nature, and especially that all sources of fatigue and ill-health should be removed as far as possible. You will, of course, relieve any constipation and cure any anæmia which may be present; advise plenty of fresh air, and let the patient stop all fatiguing occupations. The second point we have to consider is daily rest, which may be carried out by recumbency. Thirdly, correct posture and the attitude in standing, sitting, and walking. Fourthly, we have to prescribe exercises which shall strengthen the weak muscles as far as possible. And, fifthly, we devise means for the correction of the curve. So far, we have rather dealt with the treatment of the factors; and we now want to consider the treatment of the condition itself. And to do so we employ certain corrective measures, which may take the form of exercises. Sixthly, we have to discuss the question of spinal supports, and, lastly, whether there is not some more rapid measure than any which have been hitherto in vogue.

### Lying Down.

First, as to recumbency: in every case of spinal curvature it is, in my opinion, essential to give the back a certain amount of rest during the day, because in a curved back such as I have described there is always acting as a downward force the weight of the head and shoulders: and once a body is curved the tendency of weight is to increase that curvature: which is the case when a child with a curved spine is allowed to go about all day. So that the efforts which you may be making in other directions will be negatived. Secondly, the patient should be carrying out a certain number of exercises: and where the deformity is considerable it is probably the only way in which a certain amount of curvature can be corrected. Recumbency does not merely mean that the child is to lie down, but she must be in a most comfortable position, so as to relieve the strain on the back. Some curves are much improved if the patient lies on her face, particularly the lumbar curves. Dorsal curves are much better treated by placing the patient on the back. If there should be a large degree of scoliosis I should advise you to place under the dorsal region a pillow, which will have the effect of arching the chest forward, and reducing somewhat the kyphosis which is frequently an integral part of the lateral deviation. If the patient is weak in the lumbar region, when she lies down a pillow should be put under the loins to prevent the sagging strain on the muscles and ligaments. The patient should lie as a rule from one to two hours; one hour after lunch, and one in the evening. We also have to begin at the beginning and teach the child how to assume the erect attitude, and how to walk well. And you must get into the child's mind a proper sense of muscle-equilibrium; she often has no sense of balance. You can only do this by careful drilling and superintendence. People who walk badly do so because they were never taught in childhood to walk properly. Sometimes I ask children who are brought to me to stand upright, and they stand with the spine deviated, believing themselves to be straight. Then I put them in a proper upright position and ask them how they feel, and they reply "Quite crooked." You have to teach the child to assume, and then to maintain, the normal upright position.

### Muscular Weakness.

Secondly, you have to improve the nutrition of the muscles, and no doubt the best possible means is massage. Notice that I use the word "massage" without any qualifying adjective, because, after all, massage is a comparatively simple matter, and consists of certain well-recognised movements. These anybody can learn after a few short lessons, and the object is to improve the nutrition of the muscles: massage consists of kneading and squeezing, rather coarse tapping with the tips of the fingers, gentle tapping, flicking with the tips of the fingers, and finally friction. The difficulty is so to do it that you improve the condition of the muscles without wearying the patient. Some people have suitable hands for massage; others have not.

### Treatment by Apparatus.

Now we come to the exercises, which are very important. Their object is to improve the flexibility of the spine, to straighten out certain curves, and to strengthen certain muscles which require it. In a paretic case it may be very difficult to estimate which are the strong muscles and which the weak ones. In an early stage, as a rule, the weaker muscles are on the side of the convexity; they have allowed the spine to sag to the convex side. When the spine has been sagging towards one side for a considerable time, the muscles on the concave side undergo structural shortening, and that is a superadded factor. In slight cases of scoliosis I maintain that exercises, as a rule, combined with massage and rest, are quite sufficient means of treatment, without going further. But there are grades of scoliosis in which more vigorous means are called for. Early this year I went to Zurich to see the methods carried out by Dr. Schultess. He has designed a number of most ingenious machines, which certainly do exactly what is required. Of course, the best orthopædic apparatus is the human hand, but unfortunately it soon gets tired. The drawback to these machines is their expense, and a second disadvantage is that they require constant skilled attention; they cannot be left to a nurse. They carry out exercises against the resistance of weights.

### Supports.

Next, I must say a word about supports. This is a thorny and vexed subject. It may be said that no case of scoliosis can be treated alone by any one of the methods which I have advocated. In every case you must select your methods and use them in their proper combinations; therefore, to dogmatise and say that every case of scoliosis must be treated by exercises is wrong. Many cases cannot be improved by exercises or anything else; you can only prevent them getting worse. It is absolutely wrong to confine the trunk of a child in a fixed apparatus of any sort in the hope that you may improve it. The drawback to supports is that they are not actively corrective; they are merely passive agents. Yet, after all, supports have their uses. In very severe cases you may supply a support with the object of preventing the patient from getting worse; again, some patients will not exert themselves in the intervals between exercises to keep up the improvement they have gained, and in those cases supports are useful.

I must leave until another occasion my remarks about the various exercises which have been devised to correct the curvatures.

